# Viscous Sputum and Systemic Edema: Key Physical Signs for Transfusion-Related Acute Lung Injury Diagnosis During Anesthesia

**DOI:** 10.7759/cureus.99430

**Published:** 2025-12-17

**Authors:** Kosei Omasa, Yasuyuki Suzuki

**Affiliations:** 1 Anesthesiology, Kyoto University Hospital, Kyoto, JPN; 2 Research, Saiseikai Research Institute of Health Care and Welfare, Tokyo, JPN; 3 Anesthesiology, Saiseikai Matsuyama Hospital, Matsuyama, JPN; 4 Pharmacology, Ehime University Graduate School of Medicine, Toon, JPN

**Keywords:** clinical diagnosis, general anesthesia, trali, transfusion complications, transfusion-related acute lung injury

## Abstract

Diagnosing transfusion-related acute lung injury (TRALI) under general anesthesia presents unique challenges for anesthesiologists, particularly when sophisticated diagnostic resources are limited. This case demonstrates how two specific physical signs, such as viscous sputum and systemic edema, provide critical diagnostic information without advanced testing equipment.

A 74-year-old man undergoing transurethral resection of the prostate (TURP) developed acute hypoxemia and hypotension after blood transfusion. We diagnosed TRALI based on characteristic white, tenacious sputum difficult to suction and generalized erythematous swelling, distinguishing it from cardiogenic edema and concurrent transurethral resection syndrome. After stopping the transfusion and administering methylprednisolone, we transferred the patient to the high-dependency unit for mechanical ventilation. His respiratory status improved progressively, leading to successful extubation on postoperative day 2.

Viscous sputum reflects protein-rich exudate from increased pulmonary vascular permeability characteristic of TRALI pathophysiology, while systemic edema patterns indicate inflammatory capillary leak rather than hydrostatic fluid retention. These observable signs enable accurate diagnosis and appropriate management when advanced diagnostics are unavailable.

This case enables clinicians to recognize distinctive TRALI features in perioperative settings, differentiate it from cardiogenic edema using physical examination, understand the pathophysiological bases of sputum and edema patterns, and apply these skills in resource-constrained environments.

## Introduction

Transfusion-related acute lung injury (TRALI) represents a severe form of acute respiratory failure occurring during or within six hours of blood transfusion [1-3]. It is characterized by non-cardiogenic pulmonary edema resulting from immune-mediated or non-immune-mediated activation of neutrophils in the pulmonary vasculature. The Two-Hit Model is widely accepted as the pathophysiological mechanism: the first hit (e.g., surgery, inflammation) primes the neutrophils, and the second hit (e.g., bioactive lipids or antibodies in blood products) activates them, causing endothelial damage and capillary leak.

For anesthesiologists working in resource-constrained settings, diagnosing TRALI becomes particularly challenging when sophisticated diagnostic tests are unavailable, and patients cannot report subjective symptoms under general anesthesia [4-8]. The differential diagnosis primarily involves transfusion-associated circulatory overload (TACO) and cardiogenic pulmonary edema. While TRALI is managed with supportive care and typically has a favorable prognosis with recovery within 72-96 hours, distinguishing it from volume overload is crucial because the treatments are diametrically opposed (fluid restriction for TRALI vs. aggressive diuresis for TACO). The ability to distinguish TRALI from TACO relies primarily on careful observation of physical signs rather than advanced testing.

This case illustrates how specific clinical observations, particularly the character of pulmonary secretions and patterns of tissue swelling, can guide diagnosis when patients develop acute respiratory failure after transfusion during surgery. The concurrent transurethral resection syndrome adds complexity to intraoperative diagnosis, demonstrating challenging diagnostic scenarios encountered in clinical practice [9].

## Case presentation

In November 2023, a 74-year-old man (height 163.8 cm, weight 45.9 kg) with hypertension, treated with nifedipine and carvedilol, was scheduled for transurethral resection of the prostate (TURP) for benign prostatic hyperplasia under general anesthesia. He was independent in activities of daily living with no signs of clinical frailty. Preoperative transthoracic echocardiography revealed preserved left ventricular systolic function (ejection fraction 68.3%), normal wall thickness (interventricular septum 9.8 mm, posterior wall 9.8 mm), and normal diastolic function (E/e' 10.0), with no valvular abnormalities. Left atrial diameter was 24.5 mm. Preoperative ECG showed a normal sinus rhythm.

On arrival in the operating room, vital signs were stable: blood pressure 142/54 mmHg, heart rate 54 beats per minute (bpm), and SpO_2_ 99% on room air. We induced anesthesia with fentanyl 150 μg, propofol target-controlled infusion-Marsh model (effect-site target 3 μg·mL⁻¹), remifentanil continuous infusion 0.1 μg·kg⁻¹·min⁻¹, and rocuronium 40 mg. Following tracheal intubation, surgery commenced using normal saline irrigation.

Approximately 70 minutes into the surgery, we observed that the drainage fluid from irrigation had become noticeably red, suggesting significant ongoing blood loss. To assess the extent of anemia and electrolyte status, we performed an arterial blood gas analysis. Results revealed hyperchloremic metabolic acidosis (pH 7.262, pCO₂ 38.9 mmHg, bicarbonate (HCO₃⁻) 16.7 mEq·L⁻¹, chloride (Cl⁻) 116.0 mmol·L⁻¹) and progressive anemia (Hgb 8.8 g/dL; baseline 10.5 g/dL). Serum sodium was 138 mEq/L (baseline 136 mEq/L) [9,10].

Given the rapid drop in hemoglobin, the visible active bleeding, and the patient's age, we initiated a leukoreduced packed red blood cell (PRBC) transfusion to maintain physiological reserve. Within five minutes of initiating the third PRBC unit (approximately 15 mL transfused; cumulative RBC volume approx. 295 mL), acute desaturation occurred with SpO_2_ declining from 99% to 92% despite maintained ventilatory parameters. Arterial blood gas analysis at this point showed severe hypoxemia (PaO₂ 197 mmHg at fraction of inspired oxygen (FiO_2_)1.0; P/F ratio 197), satisfying the criteria for acute lung injury. Notably, despite the transfusion, hemoglobin had further decreased to 7.4 g/dL, confirming that the bleeding at the time of the initial decision (hemoglobin (Hb) 8.8 g/dL) was indeed rapid and significant. Concurrently, serum sodium rose to 144 mEq/L and Cl to 128 mEq/L.

Intraoperative recognition through physical signs

In our operating theater, portable chest X-ray and transthoracic echocardiography were not immediately available for intraoperative use. Thus, physical examination became essential. Auscultation revealed bilateral inspiratory crackles (wet rales). Most notably, we suctioned approximately 10-20 mL of white, highly viscous sputum requiring high suction pressure (>150 mmHg) from the endotracheal tube, markedly thick and difficult to remove, contrasting sharply with the pink, frothy secretions typical of cardiogenic pulmonary edema [8]. Despite a total fluid input of approximately 1400 mL (crystalloids and blood products), the patient developed prominent erythematous swelling of the face and trunk, distinct from the dependent, non-erythematous accumulation expected with volume overload.

We immediately stopped the transfusion. D-chlorpheniramine maleate 5 mg was administered intravenously as an initial consideration for allergic reaction. However, the absence of wheezing and a normal capnographic pattern made anaphylaxis unlikely. Furthermore, antibiotics and the initial dose of rocuronium had been administered over two hours prior to the event, and the timing of the respiratory collapse did not coincide with any maintenance bolus of neuromuscular blockers. Based on the distinctive sputum character and inflammatory appearance of tissue swelling, we strongly suspected transfusion-related acute lung injury [7].

**Figure 1:**
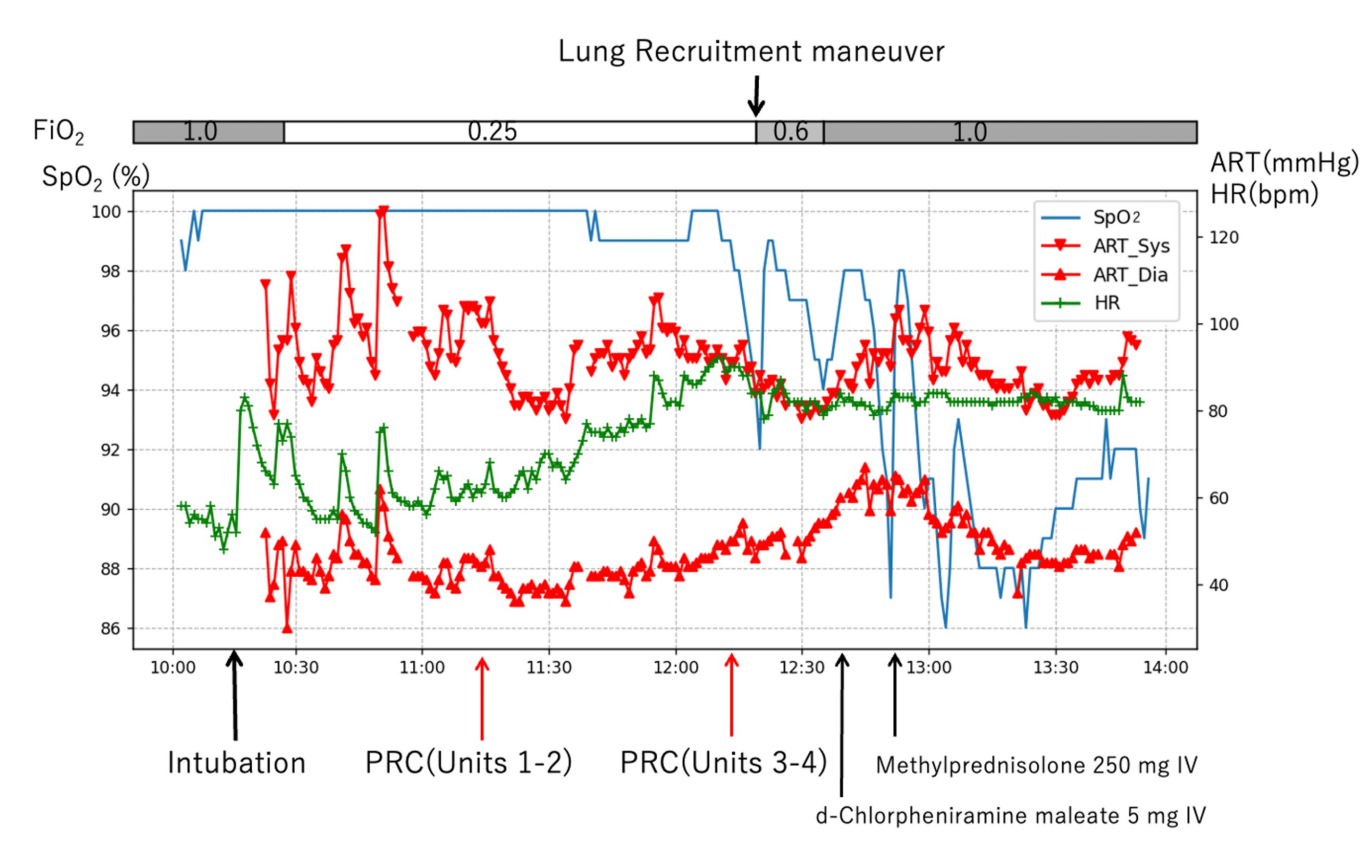
Intraoperative clinical course The patient's vital signs and interventions during surgery, with precise temporal correlations to therapeutic interventions. Blue line: peripheral oxygen saturation (SpO₂, %). Red triangles: systolic (upward) and diastolic (downward) arterial pressure (mmHg). Green line: heart rate (HR; beats per minute (bpm)). The yellow arrow indicates the timing of intubation. Orange arrows indicate packed red blood cell (PRC) transfusion initiation times. The red arrow shows the onset of symptoms (T=135 min). Purple arrows indicate medication administration: D-chlorpheniramine 5 mg IV, methylprednisolone 250 mg IV. The blue arrow indicates the lung recruitment maneuver (40 cmH₂O for 40 seconds). Note the acute deterioration within five minutes of starting the third packed red blood cell unit, demonstrating the characteristic temporal relationship of transfusion-related acute lung injury. FiO_2_: fraction of inspired oxygen; ART: arterial blood pressure

Approximately 30 minutes after starting the inciting blood product (third PRBC unit), severe hypoxemia persisted (SpO_2_ 87%) despite a lung-protective ventilation strategy (FiO_2_ 1.0, positive end-expiratory pressure (PEEP) 10 cmH_2_O, plateau pressure <30 cmH_2_O). We administered methylprednisolone 250 mg IV based on the inflammatory pathophysiology of TRALI, targeting complement activation and neutrophil-mediated lung injury (Figure 1). We completed the surgery after 159 minutes. Hemodynamic stability was maintained with continuous norepinephrine infusion of 0.05 μg·kg⁻¹·min⁻¹.

Successful high-dependency unit management and recovery

**Figure 2:**
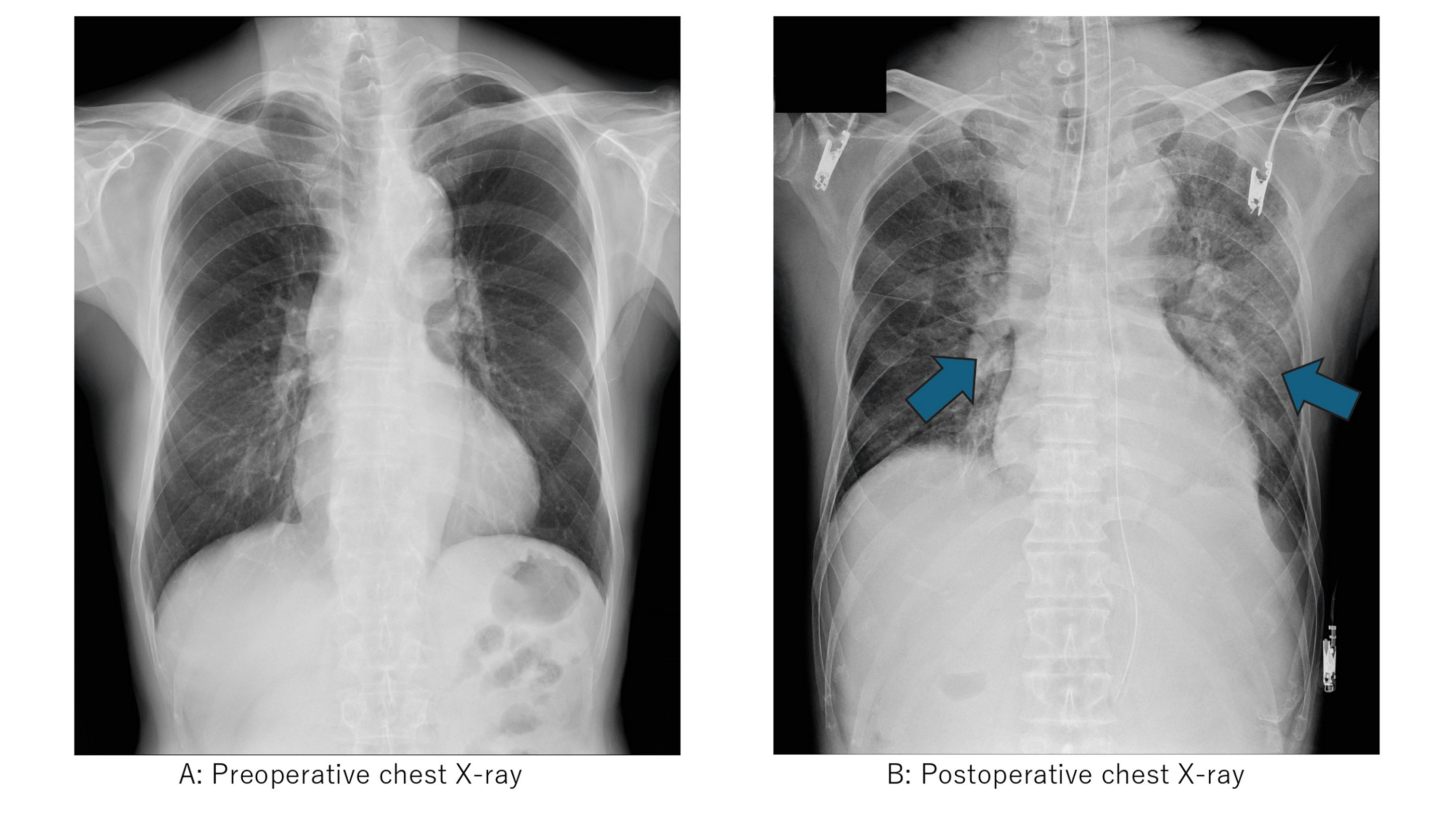
Chest radiographs (A) Preoperative posteroanterior chest radiograph: clear lung fields with normal cardiac silhouette and pulmonary vascular markings. (B) Postoperative (six hours post incident): bilateral symmetric pulmonary edema with perihilar and peripheral alveolar infiltrates consistent with transfusion-related acute lung injury (TRALI), taken when advanced imaging became available in the high-dependency unit. Blue arrows indicate areas of bilateral pulmonary infiltrates characteristic of TRALI. Note the absence of cardiomegaly or pleural effusions, supporting a non-cardiogenic etiology.

Postoperatively, we transferred the patient to the high-dependency unit. Laboratory analysis revealed NT-proBNP 85 pg/mL, ruling out heart failure. Transthoracic echocardiography revealed normal cardiac function and no signs of fluid overload, confirming our intraoperative diagnosis [11,12]. A chest X-ray showed bilateral pulmonary edema (Figure 2).

**Figure 3:**
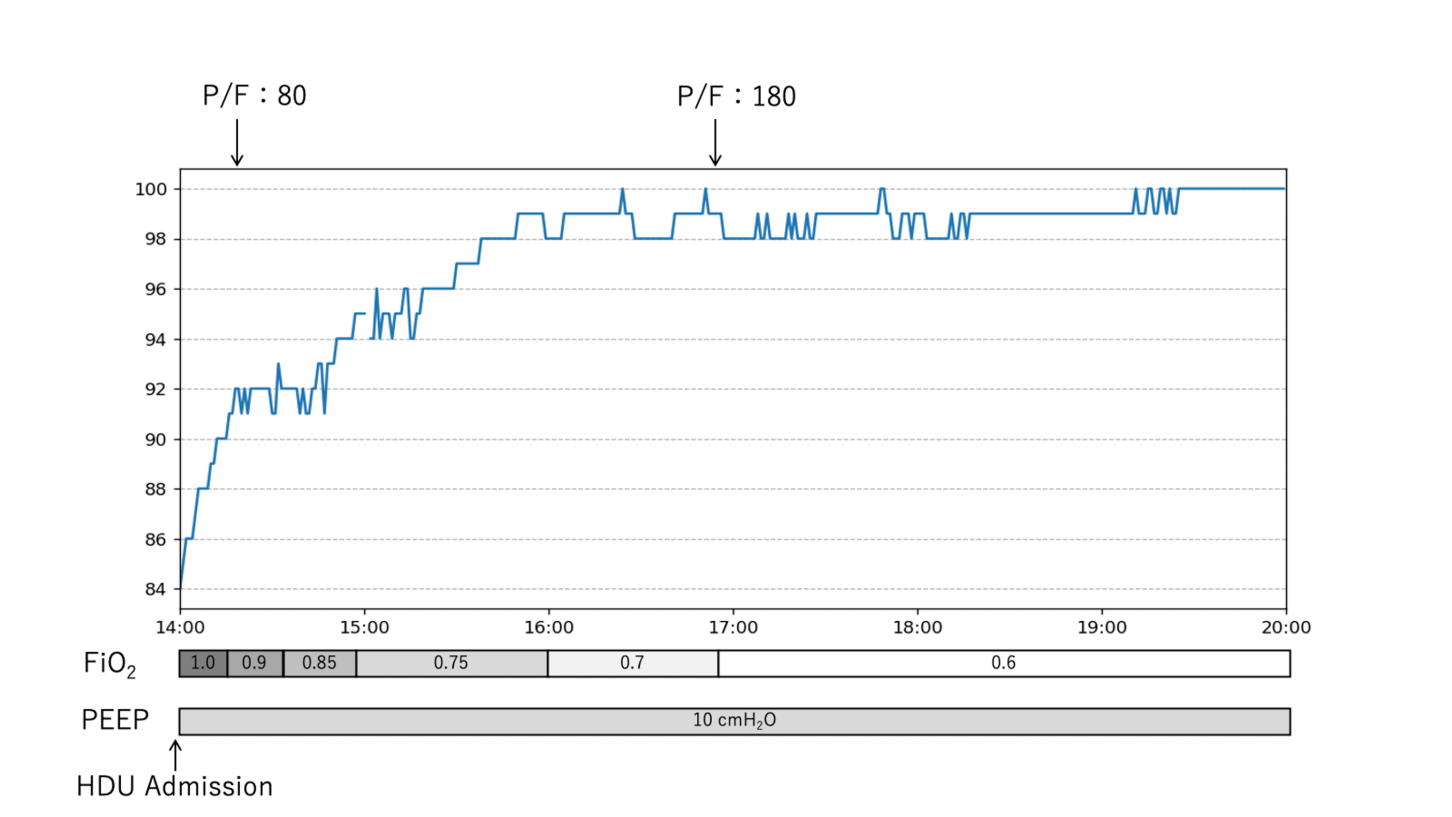
Postoperative respiratory recovery Temporal progression of respiratory parameters demonstrating a characteristic rapid improvement pattern of TRALI. PaO₂/FiO₂ ratio (mmHg) improved from 80 to 180 within three hours of high-dependency unit (HDU) admission. Bottom bars show fractional inspired oxygen (FiO₂) and positive end-expiratory pressure (PEEP, cmH₂O) settings, demonstrating progressive weaning of ventilatory support.

Respiratory status improved progressively. The oxygenation index (PaO_2_/FiO_2_) improved from 80 to 180 mmHg within three hours (Figure 3). We successfully extubated him on postoperative day 2, with complete resolution of facial and truncal swelling by day 3. He was discharged on day 7.

Subsequent investigation by the Japanese Red Cross Society conducted comprehensive antibody screening. No detectable anti-haptoglobin antibodies, anti-IgA antibodies, or anti-leukocyte antibodies, including HLA Class I, HLA Class II, HNA, and CD36, were found in blood products or patient samples. According to consensus TRALI diagnostic criteria, this case fulfilled the definition of Type I (antibody-undetectable) TRALI [13]. The patient was followed up for one year postoperatively, during which he remained asymptomatic with no respiratory sequelae, and the follow-up was subsequently concluded.

## Discussion

Diagnostic significance of physical examination in resource-constrained settings

This case provides valuable insights for anesthesiologists working in environments where sophisticated diagnostic tools may be unavailable. During surgical procedures, clinicians face unique challenges: patients cannot describe symptoms, portable chest X-rays are often impractical during ongoing surgery, and while transesophageal echocardiography may be available in some centers, transthoracic echocardiography is not routinely available in many operating rooms. In our institution, transthoracic echocardiography was unavailable in the operating room, necessitating reliance on observable physical signs for diagnosis [6,8].

Rigorous exclusion of differential diagnoses

TACO, TURP Syndrome, and Anaphylaxis

A critical aspect of this case was the differentiation from TACO, TURP syndrome, and anaphylaxis, especially given the fluid load during endoscopic surgery. First, TACO was ruled out based on objective cardiac function markers. The patient had normal preoperative diastolic function (E/e' 10.0), indicating no pre-existing diastolic heart failure prone to hydrostatic edema. Furthermore, the postoperative NT-proBNP level was 85 pg/mL, which is well within the normal range and strongly argues against significant left atrial hypertension or volume overload as the primary cause of pulmonary edema [11,12]. Second, classical TURP syndrome (hyponatremic water intoxication) was excluded by the intraoperative electrolyte trends. Instead of hyponatremia, our patient demonstrated a rise in serum sodium (from 136 to 144 mEq/L) and a marked increase in chloride (from 107 to 128 mEq/L). This hyperchloremic pattern confirms the absorption of normal saline irrigation fluid rather than hypotonic fluid. While saline absorption causes volume expansion, the absence of elevated cardiac filling pressures (normal E/e' and NT-proBNP) suggests that the pulmonary edema was not hydrostatic (cardiogenic) but rather due to increased vascular permeability. Finally, anaphylaxis was unlikely given the absence of wheezing, cutaneous flushing, or hypotension preceding the hypoxemia. Crucially, the administration of antibiotics and the initial dose of neuromuscular blockers occurred over two hours prior to the event, lacking the temporal correlation typical of IgE-mediated reactions.

Pathophysiological basis of sputum characteristics

The character of pulmonary secretions provided the most valuable diagnostic clue in this case. The white, tenacious sputum suctioned from our patient's endotracheal tube differed markedly from the pink, frothy secretions of cardiogenic edema or transfusion-associated circulatory overload. This viscous quality reflects protein-rich exudate characteristic of acute pulmonary capillary-alveolar barrier dysfunction with increased microvascular permeability in TRALI [14], containing polymorphonuclear leukocyte degranulation products, including elastase, myeloperoxidase, and cytotoxic mediators that increase secretion viscosity [3,14]. Activated neutrophils release elastase, myeloperoxidase, and DNA-histone complexes that contribute to both lung injury and the characteristic thick, difficult-to-suction consistency observed clinically [1,15]. In contrast, the frothy pink sputum of cardiogenic edema represents protein-poor transudates mixed with red blood cells from elevated hydrostatic pressures [6,8]. This pathophysiologically-based distinction, readily observable during routine endotracheal suctioning, provides immediate diagnostic information without requiring laboratory analysis.

Inflammatory edema pattern recognition in systemic capillary leak

The pattern of tissue swelling was the second critical diagnostic feature. Our patient developed generalized erythematous edema of the face and trunk, indicative of systemic capillary leak syndrome with increased transcapillary fluid flux associated with TRALI pathophysiology [4,11]. This inflammatory process involves classical and alternative complement pathway activation with C5a and C3a generation, neutrophil priming, and cytokine-mediated increases in vascular permeability that extend beyond the pulmonary circulation [15]. The erythematous appearance reflects underlying inflammatory vasodilation and increased capillary permeability, fundamentally different from cardiogenic edema, where fluid accumulation results from elevated hydrostatic pressure without primary inflammatory changes, typically presenting as dependent, non-erythematous accumulation. These findings, observable through basic inspection, guided our diagnosis despite the confounding presence of transurethral resection syndrome.

Clinical application of the Two-Hit Model

The concurrent transurethral resection syndrome added diagnostic complexity, as the Two-Hit Model explains how multiple factors can contribute to TRALI development [15]. The significant absorption of saline irrigation fluid, evidenced by the hyperchloremic metabolic acidosis (Cl⁻ 116.0 mmol·L⁻¹ at onset), likely acted as the "first hit," causing endothelial priming through systemic inflammation [15]. The subsequent blood transfusion then served as the precipitating trigger (second hit), with donor antibodies or bioactive lipids causing neutrophil activation and pulmonary vascular injury in primed endothelium [15]. This mechanistic understanding explains why the specific characteristics of sputum and inflammatory patterns suggested TRALI as the primary process, despite concurrent volume-related changes from irrigant absorption.

Practical clinical applications for technology-dependent practice

For anesthesiologists and other clinicians, this case demonstrates that sophisticated technology cannot replace fundamental clinical observation skills. In the operating room or emergency department, where diagnostic options may be limited and time is critical, recognizing these pathophysiologically-based patterns, viscous versus frothy sputum, inflammatory versus dependent edema, can mean the difference between appropriate supportive care and potentially harmful interventions like aggressive diuresis [7,11]. These pathophysiologically-based physical signs, such as viscous protein-rich secretions and inflammatory edema patterns, provide immediate diagnostic information with sensitivity and specificity comparable to invasive hemodynamic monitoring, making them particularly valuable in resource-limited environments where advanced diagnostics may be delayed or unavailable.

## Conclusions

This case highlights that in resource-constrained settings, specific physical signs can distinguish TRALI from cardiogenic edema and fluid overload. Crucially, white, viscous sputum indicates protein-rich inflammatory exudate, contrasting with the pink, frothy secretions of hydrostatic edema. Similarly, erythematous systemic edema suggests inflammatory capillary leak rather than simple volume retention. Recognizing these distinctive pathophysiological patterns allows for accurate diagnosis and appropriate supportive care even when advanced intraoperative imaging is unavailable.
